# Endoscopic injection of lauromacrogol foam sclerotherapy for rectal cavernous hemangioma: A case report

**DOI:** 10.1097/MD.0000000000038919

**Published:** 2024-07-12

**Authors:** Ling Chen, FengYu Chen, Tong Jiang, Yue Deng, GuoQing Shi

**Affiliations:** a Department of Gastroenterology, Digestive Disease Hospital, The Affiliated Hospital of Zunyi Medical University, Zunyi, Guizhou Province, China; b Fenggang County People’s Hospital , Fenggang, Guizhou Province, China.

**Keywords:** case report, cavernous hemangioma, foam sclerotherapy, lauryl alcohol, rectum

## Abstract

**Rationale:**

Rectal cavernous hemangioma is a rare, benign vascular disease that seldom causes lower gastrointestinal bleeding, characterized by a high rate of misdiagnosis and missed diagnoses. Surgical treatment is considered to be relatively effective; however, it is accompanied by certain employed in the treatment of superficial hemangioma, boasting the advantages of minimally invasive surgery, including safety, effectiveness, reduced trauma, and rapid recovery. However, there is a lack of literature regarding the application of foam sclerosing agents for gastrointestinal hemangiomas.

**Case concerns:**

We present a case of a 60-year-old male who was admitted to our hospital with a history of recurrent hematochezia for >1 year and worsening symptoms for 1 week. The patient's medical history was unremarkable.

**Diagnoses:**

Following colonoscopy, nuclear magnetic resonance imaging, computed tomography, and other examinations, the final diagnosis was rectal cavernous hemangioma.

**Interventions:**

Due to the patient’s refusal of surgery, endoscopic foam sclerotherapy using a lauromacrogol injection was performed after obtaining informed consent from the patient and their relatives.

**Outcomes:**

Post-sclerotherapy, hematochezia symptoms ceased, and no adverse reactions were observed. Two months later, colonoscopy and nuclear magnetic resonance imaging showed that the hemangioma had almost completely disappeared, with only a small amount of tumor remnants, yielding a satisfactory curative effect.

**Conclusion:**

Our findings indicate that endoscopic injection of a lauromacrogol foam sclerosing agent is a safe, effective, and minimally invasive treatment option for gastrointestinal cavernous hemangiomas.

## 1. Introduction

Gastrointestinal hemangioma has a low incidence of systemic hemangioma and is a rare cause of gastrointestinal bleeding, with a high rate of misdiagnoses and missed diagnoses.^[1]^ Surgical resection of the lesion is considered a relatively effective treatment; however, it has some limitations such as high surgical trauma, high risk, and slow recovery. Injection of a foam sclerosing agent has been successfully used in the treatment of superficial hemangioma,^[1]^ which is a minimally invasive treatment method with the advantages of safety, effectiveness, less trauma, and quick recovery. However, there are no reports on the application of foam sclerosing agents for gastrointestinal hemangiomas. A case of rectal cavernous hemangioma treated with endoscopic multipoint injection of lauromacrogol foam sclerosing agents reported satisfactory results.

## 2. Case report

**Figure 1.**
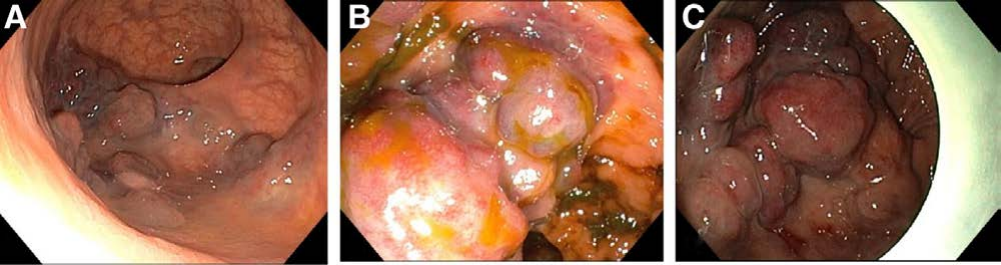
Colonoscopy before treatment: multiple purple nodular eminence was found in the rectum 6 to 10 cm from the anus, which were distributed like grape clusters, with an area of 5.0 cm × 4.0 cm.

**Figure 2.**
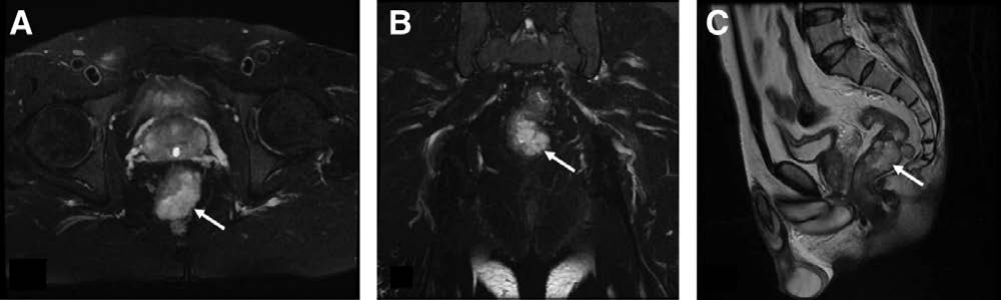
Pretreatment MRI image (T2W1): multiple irregular nodular thickening of the lower rectal wall, showing long T2 signals, and progressive enhancement on enhanced scan (white arrow). (A) Axial view; (B) coronal; (C) sagittal view.

A 60-year-old male patient was admitted to the hospital because of recurrent hematochezia for more than 1 year and aggravation for 1 week. The patient had repeated hematochezia for 1 year, which was bright red, and the amount was not high; therefore, it was not taken seriously. Hematochezia aggravated for a week, with blood stool 2 to 3 times a day, and the volume was approximately 100 mL. The patient experienced slight dizziness and fatigue, past and family history were unremarkable. Physical examination on admission revealed mild anemia with a routine blood hemoglobin level of 95 g/L. Colonoscopy revealed multiple purple nodular eminences approximately 6 to 10 cm from the rectum to the anus, which were distributed like grape clusters with an area of 5.0 cm × 4.0 cm (Fig. 1). Pelvic nuclear magnetic resonance imaging (MRI) showed multiple irregular nodular thickenings in the wall of the lower rectum 6.5 cm from the anal verge, with an upper and lower diameter of approximately 4.1 cm, presenting long T1 and T2 signals, and DW1 presented a high signal. The lesions were mostly located in the mucosa and submucosa, with involvement of the muscular layer scattered around the rectum, and progressive enhancement on an enhanced scan, which was considered a cavernous hemangioma (Fig. 2). The final diagnosis was a cavernous hemangioma of the rectum. As the patient refused surgery, endoscopic foam sclerotherapy with lauromacrogol injection was performed after informed consent was obtained from the patient and their relatives.

Preparation of lauromacrogol foam sclerosing agent (Tessari method):^[2]^ (1) connect 2 disposable syringes with a medical silicone three-way stopcock valve holder, and pay attention to the angle between the 2 syringes at 90° when connecting. (2) A certain volume of lauromacrol injection was extracted according to the liquid–gas ratio of 1 ∶ 3 (extract a certain volume of lauromacrol injection from 1 syringe, and extract 3 times the volume of lauromacrol injection air from the other syringe). The 2 syringes were then pushed back and forth 10 times (1 syringe back and forth counted as 1 injection). (3) Reduce the three-way valve channel and continue to push the injection back and forth 10 times. After injection, a stable homogeneous foam lauromacrogol foam hardener was obtained.

**Figure 3.**
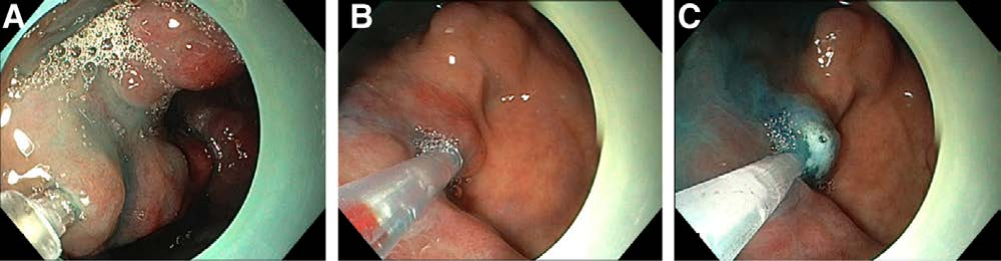
A total of 30 mL of lauromacrogol foam hardener was injected into the hemangioma under the assistance of a transparent cap through endoscope at different point.

With the assistance of a transparent cap, a lauromacrogol foam hardener was injected into the hemangioma at different points. In the past, when we treated esophageal and gastric hemangioma with lauromacrogol, 1.0 mL lauromacrogol was injected per 1.0 cm × 1.0 cm hemangioma area.^[3]^ In this case, 1.0 to 2.0 mL of lauromacrogol foam hardener was injected per 1.0 cm × 1.0 cm of the hemangioma area, so that the tumor was blue and swollen. Fractional puncture was performed on the hemangioma using a disposable endoscopic injection needle (25G, Boston Scientific). Six points are punched. After the blood return, 3 to 8 mL of foam hardener was injected at each point. A total volume of 30 mL was injected (Fig. 3). Postoperative ceftazidime prophylactic anti-infection therapy was administered for 24 hours, and the patient was instructed to maintain normal stools and avoid spicy and irritating diets.

**Figure 4.**
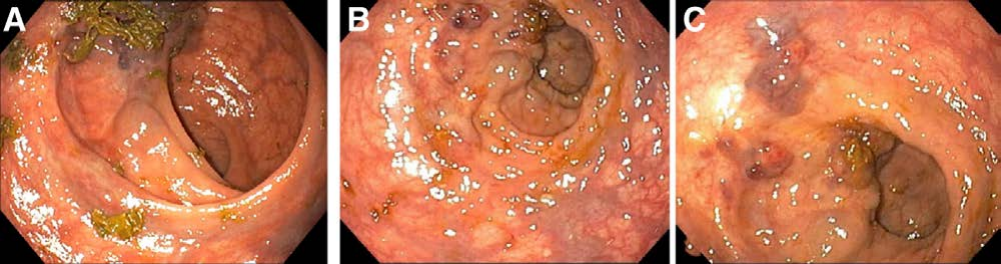
Colonoscopy 2 month after treatment: 2 months after the operation, colonoscopy showed that the hemangioma almost completely disappeared, and only a small amount of tumor remained. (A) Axial view; (B) coronal; (C) sagittal view.

**Figure 5.**
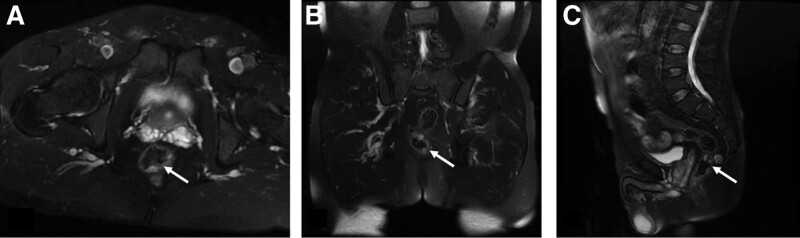
Post-treatment MRI image (T2W1): after treatment, the lesion of cavernous hemangioma in the lower rectal was significantly reduced, and the signal intensity was reduced (white arrow).

After sclerotherapy, hematochezia symptoms disappeared, and no adverse reactions such as fever, abdominal pain, or bleeding occurred. Reexamination of the colonoscopy 2 months later showed that the hemangioma had almost completely disappeared, with only a small amount of the tumor remaining (Fig. 4). Repeat MRI showed that the cavernous hemangioma of the lower rectum had a significantly reduced signal intensity after treatment (Fig. 5). After 1 year follow-up, the patient had no symptoms such as hematochezia.

## 3. Discussion

Hemangioma is a benign congenital proliferative vascular malformation that can occur in various parts of the body. According to the pathology, it can be divided into capillary, cavernous, and mixed types. Gastrointestinal hemangiomas are very rare, accounting for only 0.05% of intestinal tumors.^[4]^ Among gastrointestinal hemangiomas, 80% of rectal and sigmoid hemangiomas are cavernous, whereas the remainder are capillary type.^[5]^ Intestinal hemangiomas can originate from the mucosa, submucosa, muscularis propria, and serosal layer.^[6]^ The most common clinical symptoms of cavernous hemangioma of the rectum are recurrent painless hematochezia and iron-deficiency anemia, which may be accompanied by nonspecific symptoms such as dizziness and fatigue.^[7]^ Blood in the stool may begin in childhood and is easily misdiagnosed as internal hemorrhoids, polyps, ulcerative colitis, etc. A small number of patients (approximately 17%) may present with intestinal obstruction due to large masses or invasion of the surrounding viscera, causing abdominal or pelvic discomfort.^[8]^ Approximately 10% of the patients have no symptoms.^[9]^

Colonoscopy is the preferred treatment for rectal cavernous hemangiomas. The typical endoscopic appearance is a blue or purple nodular bulge mainly formed by dilated and congested submucosal veins. A biopsy of the lesion should be performed to avoid uncontrolled massive bleeding. CT can reveal intestinal wall thickening and abnormal density shadows, which may have a diagnostic value for hemangiomas. However, their diagnostic value is limited. MRI is superior to CT and the preferred diagnostic imaging method. MRI shows a typical heterogeneous high signal in T2 images, and “venous stone” without a signal can be seen.^[10]^

Various treatment options are available for rectal cavernous hemangiomas including drug therapy, sclerotherapy, and surgical resection. Medication can temporarily stop bleeding, but the lesion cannot be eliminated and the symptoms recur. Surgical resection can completely eradicate the lesion and achieve cure. However, some patients are reluctant to accept this treatment because of trauma, high risk, and slow recovery. Sclerotherapy has the advantages of simplicity, rapidity, low cost, good tolerance, and short hospital stays. Lauromacrogol is a sclerosing agent produced by Shaanxi Tianyu Pharmaceutical Company, China. Its chemical name is polyoxyethylene lauryl alcohol ether, an anionic surfactant cytotoxic to endothelial cells. It can dissolve red blood cells, white blood cells, and platelets, thus collapsing the tumor tissue and causing tumor necrosis. We successfully treated multiple gastroesophageal hemangiomas with endoscopic injection of liquid lauromacrogol, resulting in complete elimination of the hemangiomas.^[3]^ However, ordinary liquid lauromacrogol injections are easily diluted by blood and washed away by blood flow, which leads to insufficient contact time between the drug and the vascular wall and an incomplete contact surface, resulting in decreased efficacy. If an excessive amount of sclerosing agent is injected, the number of adverse reactions increases. However, foam sclerosing agents can prolong the contact time and increase the contact area with the vascular endothelium, thereby improving hardening efficiency and reducing the dosage of drugs, thereby reducing toxicity and side effects. Therefore, foam sclerotherapy has been widely used in the treatment of superficial hemangioma and lower extremity varicose veins and is safe and effective. However, there are no reports on the application of foam sclerosing agents for gastrointestinal hemangiomas. To explore the application of foam sclerotherapy for the treatment of gastrointestinal hemangioma, a patient with rectal cavernous hemangioma who refused surgery was treated with a multipoint injection of lauromacrogol foam sclerosing agent under endoscopy after obtaining informed consent from the patient and their relatives. Fractional puncture of the hemangioma using a 25G disposable endoscopic injection needle (Boston Scientific). Six points are punched. After the blood return, 3 to 8 mL of foam hardener was injected at each point. The patient received a total volume of 30 mL. After the operation, the hematogenous stool symptoms disappeared, and no fever, abdominal pain, bleeding, or other adverse reactions occurred. Two months later, colonoscopy and MRI showed that the hemangioma had almost completely disappeared, with only a small amount of the tumor remaining. After 1 year follow-up, the patient had no symptoms such as hematochezia and achieved good efficacy and safety.

For endoscopic foam sclerotherapy of gastrointestinal cavernous hemangiomas, we summarized the following operation points: (1) a foam sclerosing agent should be prepared with air or CO_2_. It should be used immediately after preparation and should not be stored too long; otherwise, the foam will liquefy. (2) Puncture was performed using an endoscope with a transparent endoscopic needle and a foam sclerosing agent was injected after the blood returned. Multipoint injections were administered according to the size of the hemangioma. In the past, when we treated esophageal and gastric hemangioma with lauromacrogol, 1.0 mL lauromacrogol was injected per 1.0 cm × 1.0 cm hemangioma area.^[3]^ In this case, we injected lauromacrogol foam hardener per 1.0 cm × 1.0 cm of the hemangioma area. It is advisable to make the tumor discolored and swollen during the injection. (3) Puncture can be performed using a transparent cap. The transparent cap can fix the puncture target vein and maintain the puncture field to facilitate the puncture. At the same time, transparent cap compression is one of the best hemostasis methods when bleeding is extracted during injection.^[11]^

## 4. Conclusion

In summary, endoscopy and MRI are crucial diagnostic tools for colorectal cavernous hemangioma. In comparison to drug and surgical interventions, sclerotherapy presents several advantages such as simplicity, rapidity, cost-effectiveness, good tolerance, and short hospital stays. The endoscopic injection of a lauromacrogol foam sclerosing agent for gastrointestinal cavernous hemangioma addresses the drawbacks of insufficient contact time and incomplete contact surface, thereby enhancing efficacy, improving hardening efficiency, reducing drug dosage, toxicity, and side effects. Consequently, the endoscopic multipoint injection of a lauromacrogol foam sclerosing agent demonstrates minimal invasiveness, safety, effectiveness, simplicity, economy, and fewer adverse reactions, making it a viable option for clinical application. However, this study is a case report, and the follow-up time of the patients was brief. Further case studies and longer follow-up periods are required to establish precise effects.

## Author contributions

**Data curation:** FengYu Chen, Ling Chen, Tong Jiang, Yue Deng.

**Methodology:** FengYu Chen, GuoQing Shi.

**Supervision:** GuoQing Shi.

**Writing – original draft:** Ling Chen.

**Writing – review & editing:** GuoQing Shi.
